# Mitochondrial Transplantation as a New Therapeutic Approach Against Cardiac and Renal Consequences in Male Rats With Myocardial Infarction

**DOI:** 10.1111/apha.70231

**Published:** 2026-04-25

**Authors:** María Cuesta‐Corral, Alejandro Montoro‐Garrido, Ana Romero‐Miranda, Fabián Islas, Bunty Ramchandani, Ricardo Gredilla, Joaquín Fernández‐Irigoyen, Enrique Santamaría, Beatriz Delgado‐Valero, Sara Jiménez‐González, Raquel Rodrigues Díez, María Luisa Nieto, Victoria Cachofeiro, Ernesto Martínez‐Martínez

**Affiliations:** ^1^ Departamento de Fisiología, Facultad de Medicina Instituto de Investigación Sanitaria Gregorio Marañón (IiSGM), Universidad Complutense de Madrid Madrid Spain; ^2^ Unidad de Imagen Cardíaca, Hospital General Universitario de Talavera de la Reina Toledo Spain; ^3^ Servicio de Cirugía Cardiaca Infantil, Hospital La Paz Madrid Spain; ^4^ Proteomics Platform, Navarrabiomed, Hospital Universitario de Navarra (HUN), Universidad Pública de Navarra (UPNA), IdiSNA Pamplona Spain; ^5^ Ciber de Enfermedades Cardiovasculares (CIBERCV), Instituto de Salud Carlos III Madrid Spain; ^6^ Instituto de Biología y Genética Molecular, CSIC‐Universidad de Valladolid Valladolid Spain

**Keywords:** cardiac fibrosis, endoplasmic reticulum stress, inflammation, mitochondrial transplantation, myocardial infarction, oxidative stress, renal damage

## Abstract

**Aim:**

Myocardial infarction (MI) is one of the leading causes of death worldwide. MI is associated with cardiac structural and functional alterations. Among these, cardiac fibrosis may be significantly influenced by mitochondrial dysfunction. We sought to evaluate whether the injection of functional mitochondria from healthy muscle could improve the detrimental consequences of MI.

**Methods:**

Male Wistar rats were submitted to MI through the ligature of the left anterior descending coronary artery. Animals subjected to a sham operation (the same surgical procedure without fastening of the suture that passes through the LAD) were included as a reference group (Sham). At the time of surgery, either vehicle (PBS) or isolated mitochondria (equivalent to 180 μg of mitochondrial protein in 75 μL of vehicle) were directly injected into the myocardium around the ligation to half of the animals in each group. Animals were sacrificed 4 weeks after both MI induction and the evaluation of cardiac and systolic functions.

**Results:**

Cardiac mitochondrial transplantation was able to prevent the decrease in systolic function and the development of cardiac fibrosis in MI rats. These beneficial effects were accompanied by a reduction in cardiac hypertrophy, oxidative stress, endoplasmic reticulum stress activation, and inflammatory markers. We also evaluated the effects of mitochondrial transplantation by a proteomic analysis. In addition, cardiac mitochondrial transplantation was able to prevent the development of renal alterations observed in MI rats.

**Conclusions:**

The data reveal novel mechanisms of mitochondrial transplantation effects and emerge as a novel therapeutic strategy under chronic diseases such as MI.

## 1 Introduction

Heart failure (HF) represents the end stage of cardiovascular diseases, which remain a leading cause of death worldwide. Despite major pharmacological advances, myocardial infarction (MI) is still the most prevalent cause of HF [1], largely due to the massive loss of cardiomyocytes and the subsequent structural and functional remodeling of the heart [2].

The heart is the most metabolically active organ in the body, possessing the highest mitochondrial content of any tissue that accounts for up to almost 40% of cell volume in human myocardium [3, 4]. The heart derives most of its energy from mitochondrial oxidative phosphorylation when sufficient oxygen is available [5]. During the ischemic phase of MI, mitochondrial bioenergetics are impaired, leading to reduced ATP production and increased generation of reactive oxygen species (ROS), which contribute to cardiomyocyte apoptosis and cardiac remodeling. Cardiac remodeling is characterized by an excessive production of extracellular matrix (ECM) proteins and the subsequent development of cardiac fibrosis [6]. Given the essential role of mitochondria in cellular metabolism and signaling, strategies targeting mitochondrial function may provide new opportunities for preventing post‐infarction damage and preserving cardiac contractility.

Mitochondrial transplantation has emerged as an innovative strategy to restore and preserve mitochondrial function in ischemic tissues. Previous studies have demonstrated that cardiac injection of isolated functional mitochondria from healthy tissues is safe and results in rapid mitochondrial uptake throughout cardiac tissue under non‐ischemic conditions [7]. Under ischemic experimental conditions, mitochondrial transplantation has been demonstrated to improve mitochondrial functionality and increase ATP production in the acute phase in different tissues, such as heart [8], kidneys [9], lungs [10] or brain [11]. In addition, it has been observed that mitochondrial transplantation reduces oxidative stress at the renal level [12]. Elevated ROS levels activate fibrotic signaling pathways, such as those mediated by transforming growth factor‐beta (TGF‐β), which drive the proliferation of cardiac fibroblasts and excessive collagen deposition [13]. Moreover, the loss of mitochondrial function impairs intracellular calcium handling and promotes apoptosis, further exacerbating fibrotic remodeling [14]. By restoring mitochondrial bioenergetics and reducing ROS generation, mitochondrial transplantation may attenuate the fibrotic response, limiting ECM accumulation and preserving myocardial structure and compliance.

To date, most studies investigating mitochondrial transplantation have been performed in acute ischemia–reperfusion models, where mitochondria were delivered at the time of reperfusion and outcomes are assessed in the acute phase [15]. These investigations have consistently demonstrated improvements in short‐term cardiomyocyte viability, mitochondrial respiration, and contractile function. However, the impact of mitochondrial transplantation on long‐term pathological processes—such as chronic inflammation, ECM remodeling, and fibrosis—remains largely unexplored. This knowledge gap is particularly relevant in models of permanent MI, where the absence of reperfusion leads to progressive structural deterioration and sustained multi‐organ effects. In addition, it is critical to better understand the molecular pathways underlying MI that can be modulated by mitochondrial transplantation.

In the present study, we investigated the effects of mitochondrial transplantation on chronic cardiac damage in an experimental model of MI and evaluated its impact on renal alterations associated with MI. Functional and histological analyses in heart and kidney were complemented by a proteomic approach using high‐resolution mass spectrometry to identify changes in protein expression and signaling pathways associated with mitochondrial transplantation. By integrating proteomic data with functional and histological assessments, we sought to gain further insight into molecular processes related to oxidative stress, extracellular matrix remodeling, mitochondrial homeostasis, and cell survival, thereby providing a more comprehensive evaluation of the therapeutic potential of mitochondrial transplantation in post‐MI cardiac dysfunction.

## 2 Materials and Methods

### 2.1 Animal Model

**FIGURE 1:**
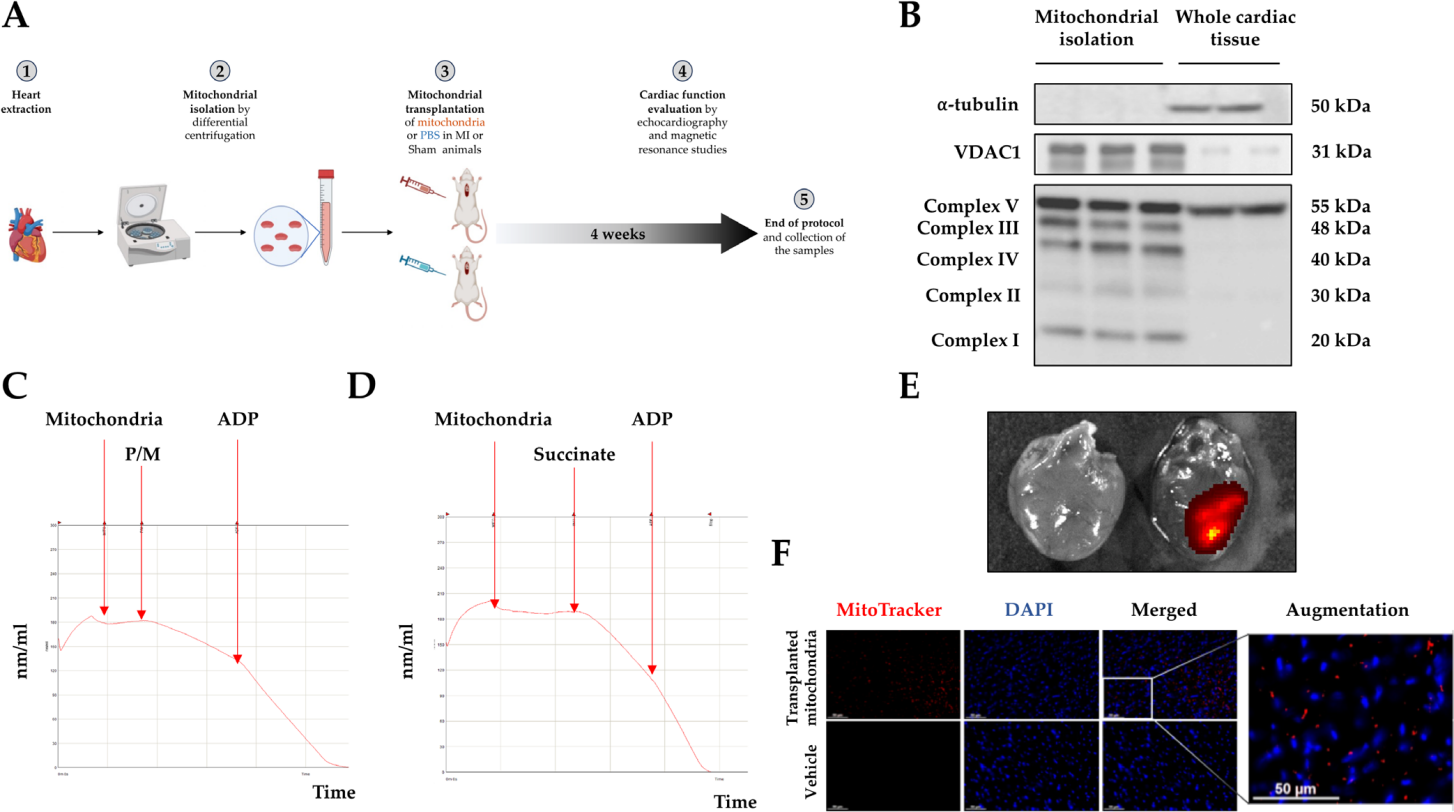
Mitochondrial isolation and viability. Schematic representation of mitochondrial isolation and animal model (A). Protein expression of ⍺‐tubulin, voltage‐dependent anion‐selective channel 1 (VDAC1), and mitochondrial complexes in samples from representative mitochondrial fractions and total heart lysates used as the positive control (B). Representative recordings of oxygen consumption in isolated mitochondria under pyruvate/malate (P/M) (C) or succinate (D) substrates in the presence of ADP (adenosine diphosphate). Optical imaging of the heart from a Sham animal (left) and from an animal that received labeled mitochondria (right) (E). Fluorescence images showing MitoTracker Deep Red FM–labeled transplanted mitochondria (red), nuclei stained with 4′,6‐diamidino‐2‐phenylindole (DAPI; blue), and merged views in heart tissue (F) from an animal that received labeled mitochondria (up) and from a Sham animal (down).

Male Wistar rats (10‐week‐old; Envigo RMS S.L., Spain) fed a standard diet were anesthetized by inhalation of isoflurane (5%; Isoflutek, Laboratorios Karizoo, Caldes de Montbuí, Barcelona, Spain) in 100% oxygen and underwent MI by ligation of the left anterior descending (LAD) coronary artery. A group of animals subjected to a sham operation (the same surgical procedure without fastening of the suture that passes through the LAD) were included as a reference group (Sham). At the time of surgery, either vehicle (PBS) or isolated mitochondria (equivalent to 180 μg of mitochondrial protein in 75 μL of vehicle) were delivered by direct injection with a 0.5 mL insulin syringe (29‐gauge needle), in four different points per rat, around the ligature of the coronary artery to half of the animals of each group. The dose of mitochondria was based on previous dose‐dependent assays [16, 17]. Four weeks after MI, animals were sacrificed (Figure 1A). The body weight was measured once a week; systolic blood pressure (SBP), and cardiac structure and function were measured the final week of the four‐week evolution period using both transthoracic echocardiography and cardiac magnetic resonance. In unrestrained rats, the SBP was estimated through tail‐cuff plethysmograph (Cibertec, Spain). At the end of the experiment, plasma and different tissues, including that of the heart and kidneys, were collected. Ethical approval for the animal study was obtained from the Animal Care and Use Committee of Universidad Complutense de Madrid and Dirección General de Medio Ambiente, Comunidad de Madrid, Spain (PROEX 349.3/21).

### 2.2 Mitochondrial Preparation and Functional Assays

Coupled mitochondria were isolated from cardiac tissue through differential centrifugation in the presence of subtilisin A, as previously described [18]. Briefly, dissected ventricles from donor rats were chopped into small pieces and homogenized with a pestle in isolation buffer (220 mM mannitol, 70 mM sucrose, 1 mM EDTA, 10 mM Tris–HCl, pH 7.4). Tissue homogenates were treated with subtilisin A (0.05 mg/mL) for 1 min at 4°C to remove extramitochondrial proteins. The nuclei and cell debris were removed by centrifugation at 700 g for 10 min. Heart mitochondria were obtained by centrifuging the supernatant twice at 8000 g for 10 min. The mitochondrial pellet was resuspended in 1 mL of PBS. At the moment of transplantation, isolated mitochondria were maintained in basal conditions in PBS, without the addition of respiratory substrates or ADP, corresponding to state 1. All the above procedures were performed at 4°C. Total mitochondrial protein was determined by the colorimetric method. The final mitochondrial suspension was maintained on ice and immediately used for oxygen consumption and transplantation.

Oxygen uptake by mitochondrial fractions was measured at 37°C by S1 Clark‐type oxygen electrode disc (Oxytherm+; Hansatec Instruments SL, UK) in 500 μL of respiration buffer (145 mM KCl, 30 mM HEPES, 5 mM MgCl2, 0.1 mM EGTA, pH 7.4). The respiratory control ratio (state 3/state 4) of the isolated mitochondria (375 μg of mitochondrial protein) was determined with complex I‐ and complex II‐ linked substrate, pyruvate/malate (250 mM each) and succinate (125 mM) respectively in the absence (state 4) followed by the presence (state 3) of ADP (2.5 mM).

### 2.3 Cardiac Function Measurement

Diastolic and systolic functions were evaluated using transthoracic echocardiography (TTE) and cardiac magnetic resonance (CMR). TTE was performed with GE Vivid‐I (General Electric Healthcare, Boston, MA, USA) portable device using a 12S‐RS transducer, and diastolic function was assessed by the early and late transmitral peak diastolic flow velocity (E and A waves), as well as the mitral annulus (lateral e' and septal e' waves) velocity. The ratios between E‐waves and A‐waves (E/A) and E‐wave by the mean peak lateral and septal e' velocities were calculated as used for assessing diastolic function. The images obtained were analyzed with EchoPAC PC version 201 software (GE Vingmed Ultrasound AS, Horten, Norway).

Cardiac magnetic resonance (CMR) imaging studies were performed with a Biospec BMT 47/40 spectrometer (Bruker, Ettlingen, Germany) in the ICTS BioImagen Center of the Universidad Complutense of Madrid, with a 12‐cm gradient system and connected to a 1025 SAM monitoring and a gating system (SA Instruments Inc., New York, NY, USA). At the beginning of the study, animals received i.p. Gd‐based contrast agent (1 mmol/kg; ProHance, Bracco Imaging, Milan, Italy) [19]. Systolic function was evaluated by left ventricle ejection fraction (LVEF) and deformation parameters such as global longitudinal strain (GLS) with the semi‐automatic tool, feature tracking (FTMR) using steady‐state balanced free precession (SSFP) sequences in left ventricle (LV) short axis and apical planes of 2‐chambers, 3‐chambers, and 4‐chambers; to obtain LV GLS. After selecting the apical views or short axis, the software automatically detects the epicardial and endocardial borders to perform myocardial tracking. If needed, manual correction is performed to improve the tracking. The software then quantifies the LV GLS. LV mass was assessed by measuring wall thickness at end‐diastole on short‐axis images. Infarct size was calculated by manually delineating regions of interest (ROI) in each slice that showed an infarcted area and then summing the volumes of each ROI to obtain the total infarct volume. All image analysis was performed using Segment version 3.0 (https://medviso.com/segment/).

Image acquisition and subsequent image analyses for TTE and CMR imaging were performed by investigators blinded to the experimental groups.

### 2.4 Mitochondria Uptake

After mitochondrial isolation, mitochondria were stained with a red fluorescence dye (MitoTracker Deep Red FM; Fisher Scientific Inc) for 30 min at 37°C. Following mitochondrial labeling, the mitochondria were injected into the hearts of Sham animals as previously described. For visualization of the mitochondria, an ex vivo analysis was performed on the heart and one kidney from a control animal (without labeled mitochondria) and from a Sham animal with mitochondria labeled with MitoTracker Deep Red. Optical imaging studies were acquired using the IVIS Lumina Series III system (PerkinElmer) with an excitation/emission filter of 620/670 nm, binning 8, Auto exposure, and f‐stop 4. A circular ROI of 0.30 cm in diameter was subsequently defined for all organs (Living Image 4.8.0), and the Maximum Radiant Efficiency was obtained.

In addition, cardiac and renal tissue were cryopreserved. 14 μm sections were visualized in a fluorescent laser scanning microscope (40× objective in a Leica DMI 3000 microscope; Leica AG, Germany).

### 2.5 Morphological and Histological Evaluation

Cardiac and renal tissue samples were dehydrated, embedded in paraffin, and cut into 4 μm thick sections. The sections were stained with picrosirius red to detect collagen fibers. The area of interstitial fibrosis was identified as the ratio of interstitial fibrosis or collagen deposition to the total tissue area after excluding the vessel area (and glomerular area in renal sections) from the region of interest. For each sample, 10 to 15 fields were analyzed with a 40× objective under transmitted light microscopy (Leica DM 2000; Leica AG, Germany). Cardiomyocytes along the left ventricle (60–80 per animal) with visible nuclei and intact cellular membranes were chosen for the determination of the cross‐sectional area in hematoxylin–eosin staining sections with a 40× objective under transmitted light microscopy. Quantitative analysis was performed using an analysis system (Leica LAS 4.3; Leica AG, Germany).

### 2.6 Measurement of Cardiac Reactive Oxygen Species Levels

To evaluate superoxide anion (O_2_^−^) levels, cardiac and renal sections (14 μm) were incubated with dye dihydroethidium (DHE; 2 × 10^−6^ M) for 30 min at 37°C. DHE is oxidized by superoxide and exhibits red fluorescence. The fluorescent signals were viewed using a fluorescent laser scanning microscope (40× objective in a Leica DMI 3000 microscope). Quantitative O_2_ levels analysis was performed using an image analyzer (Leica LAS 4.3). Three sections per animal were quantified and averaged for each experimental condition. The mean fluorescence densities in the target region were analyzed. The results are expressed as n‐fold increases over the values of the control group in arbitrary units.

### 2.7 Western Blot Analysis

40 μg of total proteins were separated by SDS‐PAGE on 4%–20% polyacrylamide CriterionTM TGX Stain‐FreeTM Precast Gels (BioRad, California, USA) and transferred to nitrocellulose membranes with the Trans‐Blot Turbo Transfer System. Membranes were probed with primary antibodies for 2,4‐dienoyl‐CoA Reductase 1 (DECR1; Novus Biologicals, Centennial, CO, USA; Ref: NBP1‐33103; dilution 1:500), activating transcription factor 4 (ATF4; Proteintech, Rosemont, IL, USA; Ref: 10835‐1‐AP; dilution 1:1000), activating transcription factor 6 alpha (ATF6α; Santa Cruz, Dallas, TX, USA; Ref: sc‐166659; dilution 1:250), binding immunoglobulin protein (BiP; BD Biosciences, Madrid, Spain; Ref: 610978; dilution 1:1000), CCAAT‐enhancer‐binding protein homologous protein (CHOP; Cell Signaling Technology, Danvers, MA, USA; Ref: 2895S; dilution 1:500), collagen I (Col I; Calbiochem, San Diego, CA, USA; Ref: 234167; dilution 1:500), collagen IV (Col IV; Santa Cruz Biotechnology, Dallas, TX, USA; Ref: sc‐398655; dilution 1:1000), Cellular Communication Network Factor 2 (CCN2; Abcam, Cambridge, UK; Ref: ab6992; dilution 1:500), eukaryotic translation initiation factor 3 subunit D (eIF3d; Abcam, Cambridge, UK; Ref: ab155419; dilution 1:500), high mobility group box 1 (HMGB1; Abcam, Cambridge, UK; Ref: ab18256; dilution 1:500), mitofusin 1 (MFN1; Abcam, Cambridge, UK; Ref: ab57602; dilution 1:1000), peroxisome proliferator‐activated receptor gamma coactivator 1‐alpha (PGC‐1α; Abcam; Cambridge, UK; Ref: ab188102; dilution 1:1000), transforming growth factor β (TGF‐β; Santa Cruz Biotechnology, Dallas, TX, USA; Ref: sc‐130348; dilution 1:1000) and total OXPHOS (CI‐NDUFB8, CII‐SDHB, CIII‐UQCRC2, CIV‐MTCO1 and CV‐ATP5A; Abcam, Cambridge, UK; Ref: ab110413; dilution 1:1000) and α‐tubulin (Sigma‐Aldrich, St. Louis, MO, USA; Ref: T5168; dilution 1:5000). Stain‐free and voltage‐dependent anion‐selective channel 1 (VDAC1; Abcam, Cambridge, UK; Ref: ab15895; dilution 1:1000) were used as loading controls for total or for mitochondrial protein, respectively. The signals were detected using the ECL system (Millipore, Burlington, MA, USA). The results are expressed as n‐fold increases over the values of the control group in arbitrary densitometric units.

### 2.8 Retrotranscription and Real‐Time PCR

Total RNA was isolated using Trizol Reagent (Fisher Scientific Inc., Waltham, MA, USA) and was reverse‐transcribed into cDNA using the High‐Capacity cDNA Reverse Transcription Kit (Fisher Scientific Inc., Waltham, MA, USA). Quantitative PCR analysis was performed with SYBR green PCR technology (Fisher Scientific Inc., Waltham, MA, USA). Quantification of mRNA levels was performed by real‐time PCR using the 2^−ΔΔCt^ method. Data were normalized to 18S ribosomal RNA (18S). The list of primers used in the study is presented in Table S1.

### 2.9 Circulating Parameters

Plasma circulating levels were measured using a specific quantitative sandwich enzyme immunoassay for aldosterone (Cayman Chemical, Ann Arbor, MI, USA; Ref: 501090), CCL2 and IL‐4 (R&D Systems) following the manufacturer's instructions.

### 2.10 Proteomic Analyses

Samples were homogenized in a lysis buffer containing 8 M urea, 2 M thiourea, 50 mM dithiothreitol, 50 mM ABC. Lysates were centrifuged at 20 000 g (1 h, 15°C), and the resulting supernatant was quantified with the Bradford assay kit (BioRad, Barcelona, Spain). Protein digestion was performed with 20 μg of protein. Proteins were reduced with DTT (final concentration of 20 mM; room temperature, 30 min), alkylated with iodoacetamide (final concentration of 30 mM; room temperature, 30 min in the dark), diluted to 0.9 M with ABC and digested with trypsin (Promega, Madison, WI, USA; 1:20 w/w enzyme protein ratio, 18 h, 37°C). Protein digestion was interrupted by acidification (pH < 6, acetic acid). Purification and concentration of peptides was performed using C18 Zip Tip Solid Phase Extraction (Millipore). Finally, peptide fraction was dried down in a Speed‐Vac system.

Dried‐down peptide samples were reconstituted with 2% ACN‐0.1% FA (Acetonitrile‐Formic acid), spiked with internal retention time peptide standards (iRT, Biognosys), and quantified by NanoDropTM spectrophotometer (ThermoFisher Sci.) prior to LC–MS/MS analysis using an EVOSEP ONE system coupled to an Exploris 480 mass spectrometer (Thermo Fisher Sci.). Peptides were resolved using a C18 Performance column (75 μm × 15 cm, 1.9 μm particles; Evosep) with a predefined Xcalibur Whisper100 20 SPD (58 min, IonOpticks Aurora Elite, EV1112) method. Peptides were ionized using 1.6 kV spray voltage at a capillary temperature of 275°C. Sample data were acquired in data‐independent acquisition (DIA) mode with full MS scans (scan range: 400 to 900 *m/z*; resolution: 60 000; maximum injection time: 22 ms; normalized AGC target: 300%) and 24 periodical MS/MS segments applying 20 Th isolation windows (0.5 Th overlap: resolution: 15000; maximum injection time: 22 ms; normalized AGC target: 100%). Peptides were fragmented using a normalized HCD collision energy of 30%.

Mass spectrometry data files were analyzed using Spectronaut (Biognosys) by direct DIA analysis (dDIA). MS/MS spectra were searched against the Uniprot proteome reference rat using standard settings. Enzyme was set to trypsin in a specific mode. Carbamidomethyl (C) was set as a fixed modification, and oxidation (M), acetyl (protein N‐term), deamidation (N), and Gln‐ > pyro‐Glu were set as variable modifications for total protein analysis.

The obtained quantitative data for total protein were exported to Perseus software (version 1.6.15.0) [20] for statistical analysis and data visualization. For total protein analysis, unpaired Student's *t*‐test was used for direct comparisons. Statistical significance was set at *p*‐value lower than 0.05 in all cases and a 1% peptide FDR threshold was considered. Differentially expressed proteins were considered significant when their absolute fold change was below 0.77 (downregulated proteins) and above 1.3 (up‐regulated proteins) in linear scale. Differential proteins were functionally analyzed using the Metascape tool [21] through gene ontology (GO) and Reactome databases using default settings (min. overlap: 3, min. enrichment: |1.5|, *p* < 0.05).

MS data and search results files were deposited in the Proteome Xchange Consortium via the JPOST partner repository (https://repository.jpostdb.org, accessed on 22 January 2025) [22] with the identifier PXD060061 for ProteomeXchange and JPST003572 for jPOST (For reviewers: https://repository.jpostdb.org/preview/18636638476790f59085593; Access key: 4703).

### 2.11 ATP Determination Kit

Total cardiac ATP levels were assayed in frozen tissue using the ATP determination kit (Molecular Probes, Eugene, OR; Ref: A22066) following the manufacturer's instructions.

### 2.12 Statistical Analysis

The sample size was calculated considering a significance level (*α*) of 0.05, a two‐tailed test, and a desired statistical power of 0.8. using the free webpage GRANMO (https://www.datarus.eu/en/applications/granmo/). Ten animals were included in each group. Variables are expressed as mean ± standard error of the mean (SEM). Normality of distributions was verified by means of the Kolmogorov–Smirnov test. Differences among groups were analyzed using two‐way ANOVA followed by Bonferroni's multiple comparisons test. A value of *p* < 0.05 was used as the cut‐off value for defining statistical significance. Outliers were eliminated from the database once they were identified using the *Z*‐score method. Data analysis was performed using the statistical program GraphPad Prism 8 (San Diego, CA, USA).

## 3 Results

### 3.1 Mitochondrial Isolation and Oxygen Consumption From Donor Animals

After isolation from heart of healthy donor rats (Figure 1A), we tested the purity of the mitochondrial fractions. The different complexes of the mitochondrial electron transport chain and VDAC1, a major component of the outer mitochondrial membrane, were detected in the isolated mitochondrial fractions. Moreover, mitochondria were isolated during the first step of the procedure in the presence of subtilisin A in order to eliminate extramitochondrial proteins. We did not detect the presence of the cytoplasmic protein ⍺‐tubulin in mitochondrial fractions, confirming the purity of the isolated mitochondria (Figure 1B). In order to perform mitochondrial transplantation, tightly coupled mitochondria are required. We evaluated mitochondrial functionality assessing mitochondrial oxygen consumption using complex I‐ and complex II‐linked substrates. Isolated mitochondria displayed high respiratory control ratio (RCR; state 3 divided by state 4 oxygen consumption) with both pyruvate‐malate (Figure 1C) and succinate (Figure 1D), indicating that energy transduction in the mitochondrial preparations was well preserved. As shown in Figure 1E, 2 h after the injection of the labeled mitochondria, their internalization can be observed in the hearts of the transplanted animals. Microscopic examination revealed a positive fluorescent labeling in the heart of the animals that received the transplantation of labeled mitochondria, whereas no signal was detected in Sham animals (Figure 1F), confirming the uptake of transplanted mitochondria in the heart of the animals.

### 3.2 Mitochondrial Transplantation Improves Cardiac Function and Remodeling in MI

MI promotes structural and functional alterations in the LV. We first assessed whether mitochondrial transplantation could influence the progression of cardiac remodeling and preserve myocardial function. Body weight and systolic blood pressure (SBP) were similar in all groups studied (Table S2). Echocardiographic and magnetic resonance data showed that MI animals presented an increase in LV mass that was accompanied by an increase in end‐diastolic diameter (EDD) (Table S2). Mitochondrial transplantation improved these morphological changes in MI animals (Table S2).

**FIGURE 2:**
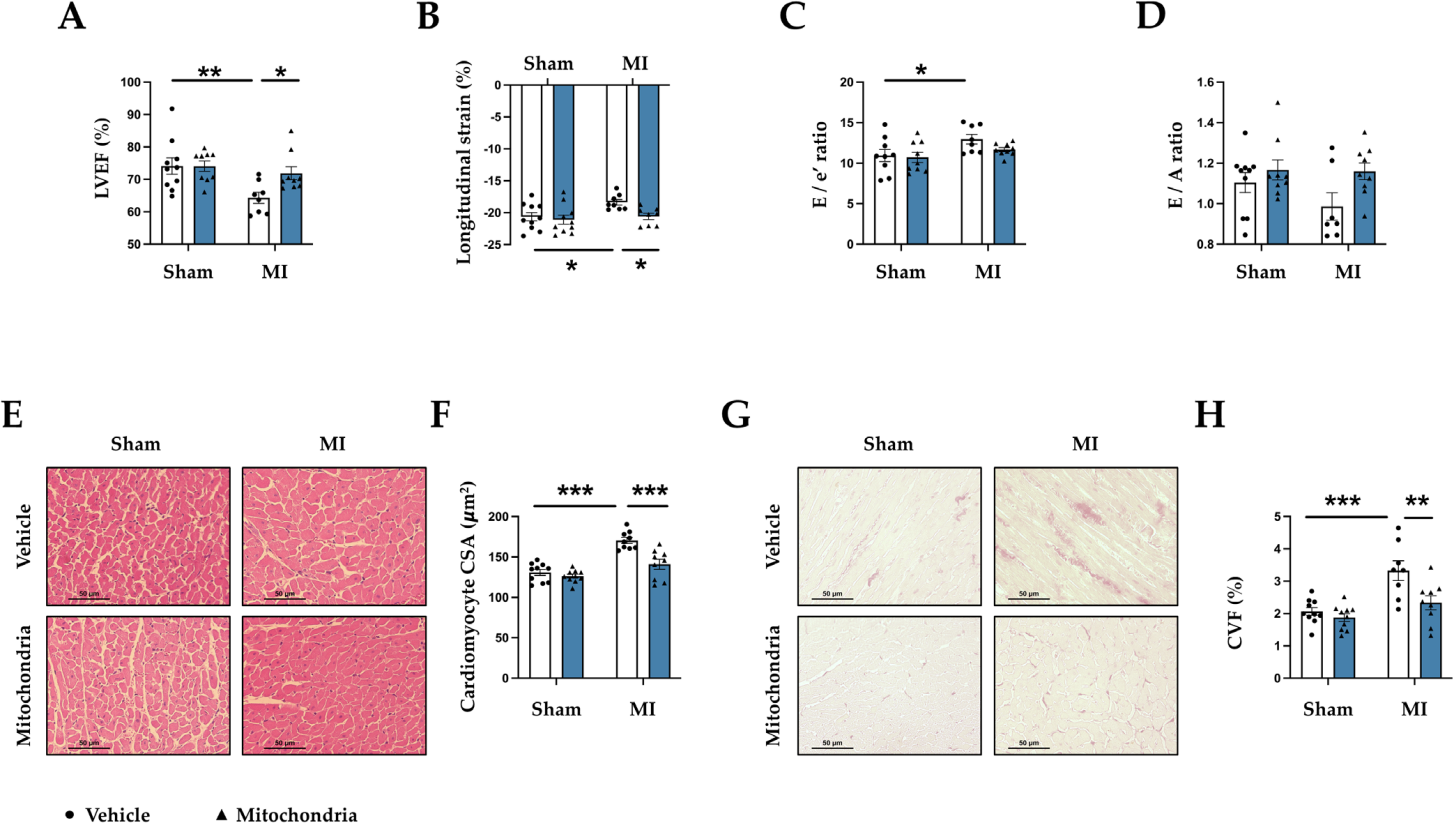
Mitochondrial transplantation improves cardiac function and remodeling in MI animals. Effects of mitochondrial transplantation on (A) left ventricle ejection fraction (LEVF); (B) longitudinal strain quantification; (C) E‐wave and e' ratio (E/e'); (D) E‐wave and A‐wave ratio (E/A); (E) representative microphotographs of cardiac sections stained with hematoxylin–eosin (magnification 40×); (F) quantification of cardiomyocytes cross‐sectional area (CSA); (G) representative microphotographs of cardiac sections stained with picrosirius red (magnification 40×) and; (H) quantification of collagen volume fraction (CVF) in control rats (Sham) and rats submitted to myocardial infarction (MI). Bars graphs (blue: Mitochondrial transplantation; white: Respective controls) represent the means ± SEM of 7–10 animals with individual points for each rat. Statistics were performed using two‐way ANOVA followed by Bonferroni's multiple comparisons test. *p < 0.05; **p < 0.01; ***p < 0.001.

Functional analyses using SSFP sequences and FTMR were performed. MI rats exhibited significantly reduced systolic left ventricular function, as assessed by LVEF and GLS (Figure 2A,B). In addition, functional impairment in diastolic function such as a significant increase in E/e' ratio (Figure 2C) and non‐significant differences in E/A ratio were found in MI rats (Figure 2D). These effects were ameliorated 4 weeks after mitochondrial transplantation in absence of differences of infarct size (Figure 2A–D) (Table S2). In addition, mitochondrial transplantation prevented the increase in LV mass (Table S2) and in cardiomyocyte cross‐sectional area observed in MI rats (Figure 2E,F). Furthermore, the increase in interstitial fibrosis observed in MI rats was reduced in mitochondrial transplanted MI animals (Figure 2G,H).

**FIGURE 3:**
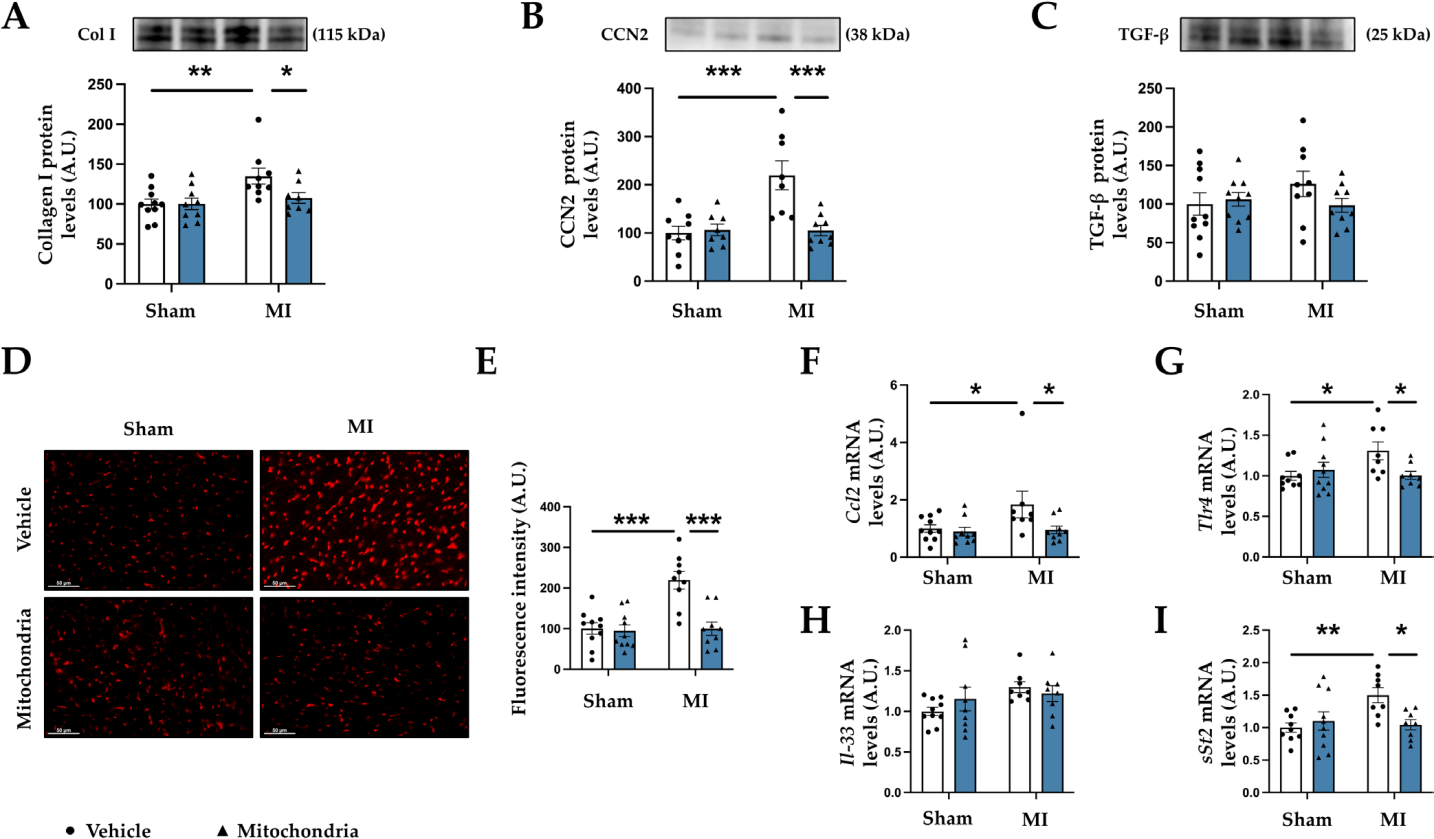
Mitochondrial transplantation improves extracellular matrix proteins, oxidative stress, and inflammation in MI animals. Effects of mitochondrial transplantation on protein levels of (A) collagen I; (B) cellular communication network factor 2 (CCN2); (C) transforming growth factor‐β (TGF‐β). (D) Representative microphotographs of cardiac sections labeled with the oxidative dye dihydroethidium (magnification 40×). (E) Quantification of cardiac superoxide anion levels. Gene expression of (F) chemokine (C‐C motif) ligand 2 (CCL2); (G) toll‐like receptor 4 (TLR4); (H) interleukin‐33 (IL‐33) and; (I) IL‐33 soluble receptor (sST2) in control rats (Sham) and rats submitted to myocardial infarction (MI). Bars graphs (blue: Mitochondrial transplantation; white: Respective controls) represent the means ± SEM of 8–10 animals with individual points for each rat. Statistics were performed using two‐way ANOVA followed by Bonferroni's multiple comparisons test. Data was normalized by stain‐free or 18S for protein levels or gene expression, respectively. *p < 0.05; **p < 0.01; ***p < 0.001.

Complementary analyses showed that mitochondrial transplantation ameliorated the increase in ECM proteins such as collagen I observed in MI animals (Figure 3A). Similarly, MI was followed by a significant increase in the profibrotic mediator CCN2 (Figure 3B) and non‐significant differences in TGF‐β protein levels (Figure 3C). These changes were also prevented by mitochondrial transplantation. A similar pattern was observed at mRNA levels. At 4 weeks post‐mitochondrial transplantation, MI animals improved collagen I, TGF‐β, and fibronectin gene expression without changes in CCN2 (Figure S1).

### 3.3 Mitochondrial Transplantation Improves Cardiac Oxidative Stress and Inflammation in MI

Given that oxidative stress and inflammation are central drivers of post‐MI injury and remodeling, we next investigated whether mitochondrial transplantation modulates these pathogenic processes in the infarcted myocardium. MI animals presented increased superoxide anion presence at the cardiac level, an effect avoided by administering functional mitochondria (Figure 3D,E). This effect appears independent of antioxidant defense, as no changes in SOD1 or SOD2 mRNA levels were observed in any of the groups studied (Figure S1).

Concerning inflammatory response, MI animals showed an increase in the pro‐inflammatory marker, chemokine (C‐C motif) ligand 2 (CCL2) (Figure 3F) together with an increase in the mRNA levels of Toll‐like receptor 4 (TLR4) (Figure 3G). In addition, MI produced an alteration in Interleukin (IL)‐33/ST2 axis (Figure 3H,I), promoting an increase in the soluble receptor (sST2) without changes in IL‐33 or the transmembrane receptor (ST2L) (Figure S1). Mitochondrial transplantation was able to prevent the increase in the inflammatory markers observed in MI animals (Figure 3F–I).

### 3.4 Mitochondrial Transplantation Improves Endoplasmic Reticulum Stress Activation and Mitochondrial Bioenergetics Observed in MI

**FIGURE 4:**
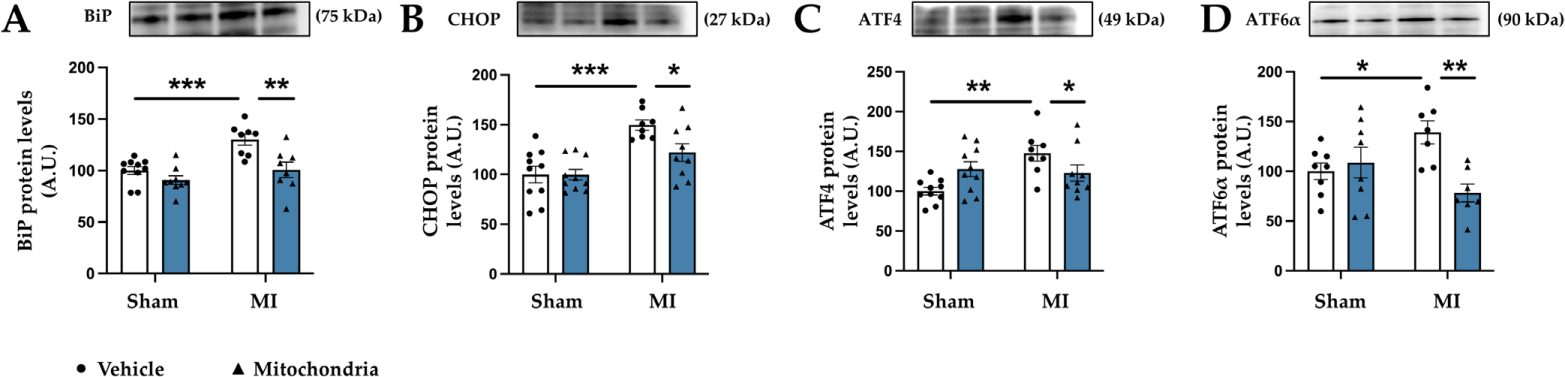
Mitochondrial transplantation improves endoplasmic reticulum stress activation in MI animals. Protein levels of (A) binding immunoglobulin protein (BiP); (B) CCAAT‐enhancer‐binding protein homologous protein (CHOP); (C) activating transcription factor 4 (ATF4); (D) activating transcription factor 6 alpha (ATF6α) in control rats (Sham) and rats submitted to myocardial infarction (MI). Bars graphs (blue: Mitochondrial transplantation; white: Respective controls) represent the means ± SEM of 7–10 animals with individual points for each rat. Statistics were performed using two‐way ANOVA followed by Bonferroni's multiple comparisons test. Protein levels were normalized by stain‐free. *p < 0.05; **p < 0.01; ***p < 0.001.

We explored the effects of mitochondrial transplantation on endoplasmic reticulum (ER) stress. After MI an increase in cardiac protein levels of the chaperone BiP, a marker of ER stress, was observed. This increase was attenuated by mitochondrial transplantation (Figure 4A). Studies of the pathways involved in the ER stress activation revealed that MI promoted an increase in CHOP (Figure 4B), as well as in ATF4 (Figure 4C) and ATF6⍺ (Figure 4D) protein levels. The injection of functional mitochondria within the heart at the moment of the MI induction prevented the activation of these downstream proteins (Figure 4B–D).

### 3.5 Proteomic Alterations in MI Animals

To obtain a global overview of the molecular pathways affected by MI and modulated by mitochondrial transplantation, we performed an unbiased proteomic analysis. To summarize the main findings, the proteomic analysis identified broad MI‐induced dysregulation of mitochondrial, cytoskeletal, and stress‐response proteins and demonstrated that mitochondrial transplantation partially restored these molecular signatures toward the profile of control animals.

**FIGURE 5:**
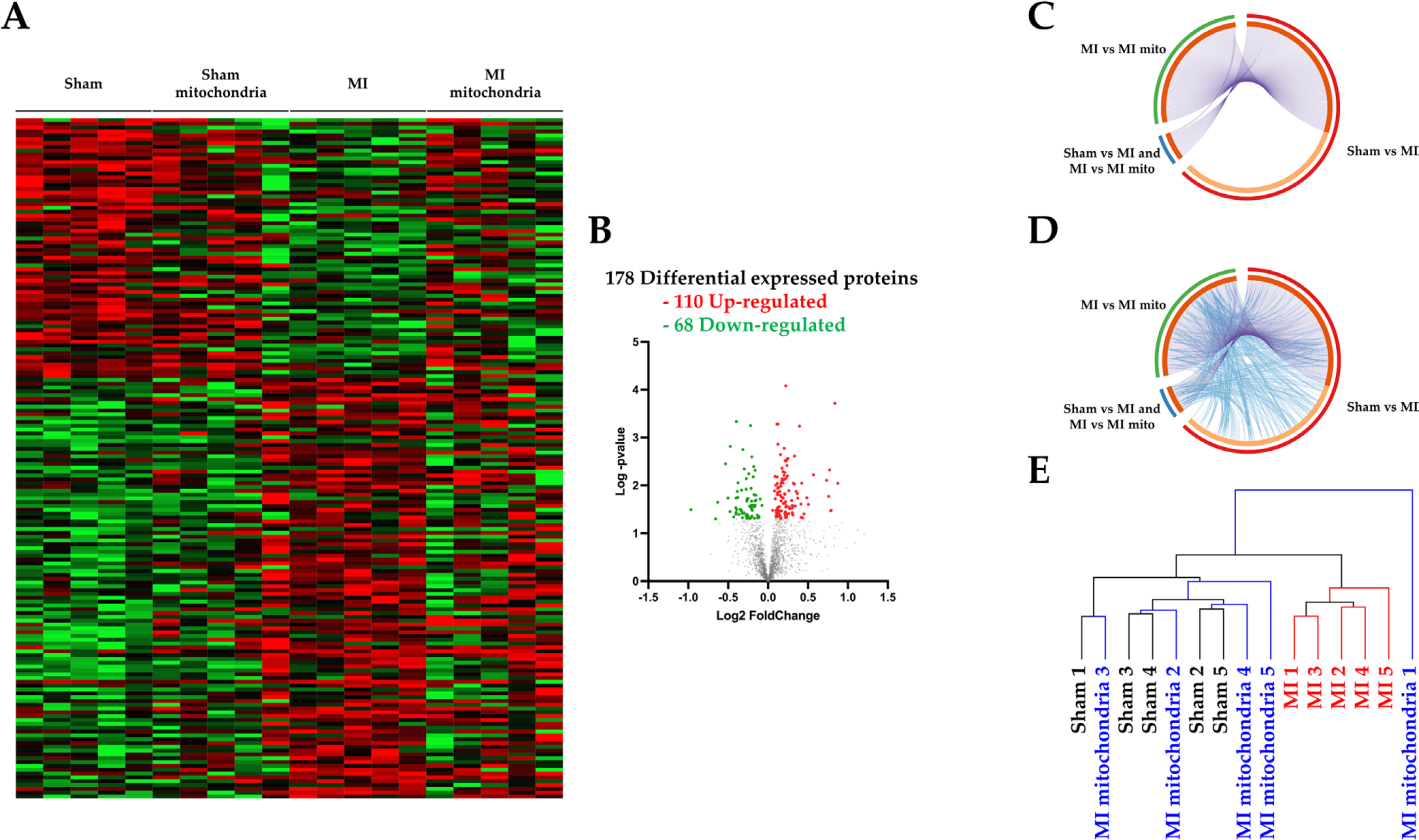
Effects of mitochondrial transplantation on proteome in MI animals. (A) Heatmap representing differential proteins expressed at the cardiac level in the animals. (B) Volcano plots represent the dysregulated proteins induced by MI. (C) Circos‐plot represents the deregulated proteome shared across the MI and mitochondrial transplantation (purple lines), and (D) the functional overlap (blue lines) between the dysregulated proteomes. (E) Clustering analyses between control rats (Sham), rats submitted to myocardial infarction (MI), and MI animals with mitochondrial transplantation.

Four weeks post‐MI, and mitochondrial transplantation, a proteomic analysis was performed in 5 heart samples of each group. Among more than 1800 proteins identified, 295 proteins were differentially expressed, taking into consideration a cutoff point of *p* < 0.05 (Figure 5A and Figure S2). MI altered the expression of 178 proteins as compared with Sham animals (Figure 5B and Table S3). Functional analyses revealed that the mainly disrupted bio functions altered by MI were a cellular response to stress, neutrophil granulation, and signaling by Rho‐GTPases (Figure S3A), the main alterations being at subcellular level related to focal adhesion, secretory granule lumen, actin cytoskeleton, but also mitochondrial matrix (Figure S3B). Animals subjected to mitochondrial transplantation differentially expressed 139 proteins (Table S3). The overlap of deregulated proteome between Sham, MI, and MI‐transplanted animals is shown in Figure 5C. Functional analyses revealed an extensive functional overlap across the analyzed samples (Figure 5D). In this sense, clustering analyses showed that mitochondrial transplanted rats are close to control animals (Figure 5E).

**FIGURE 6:**
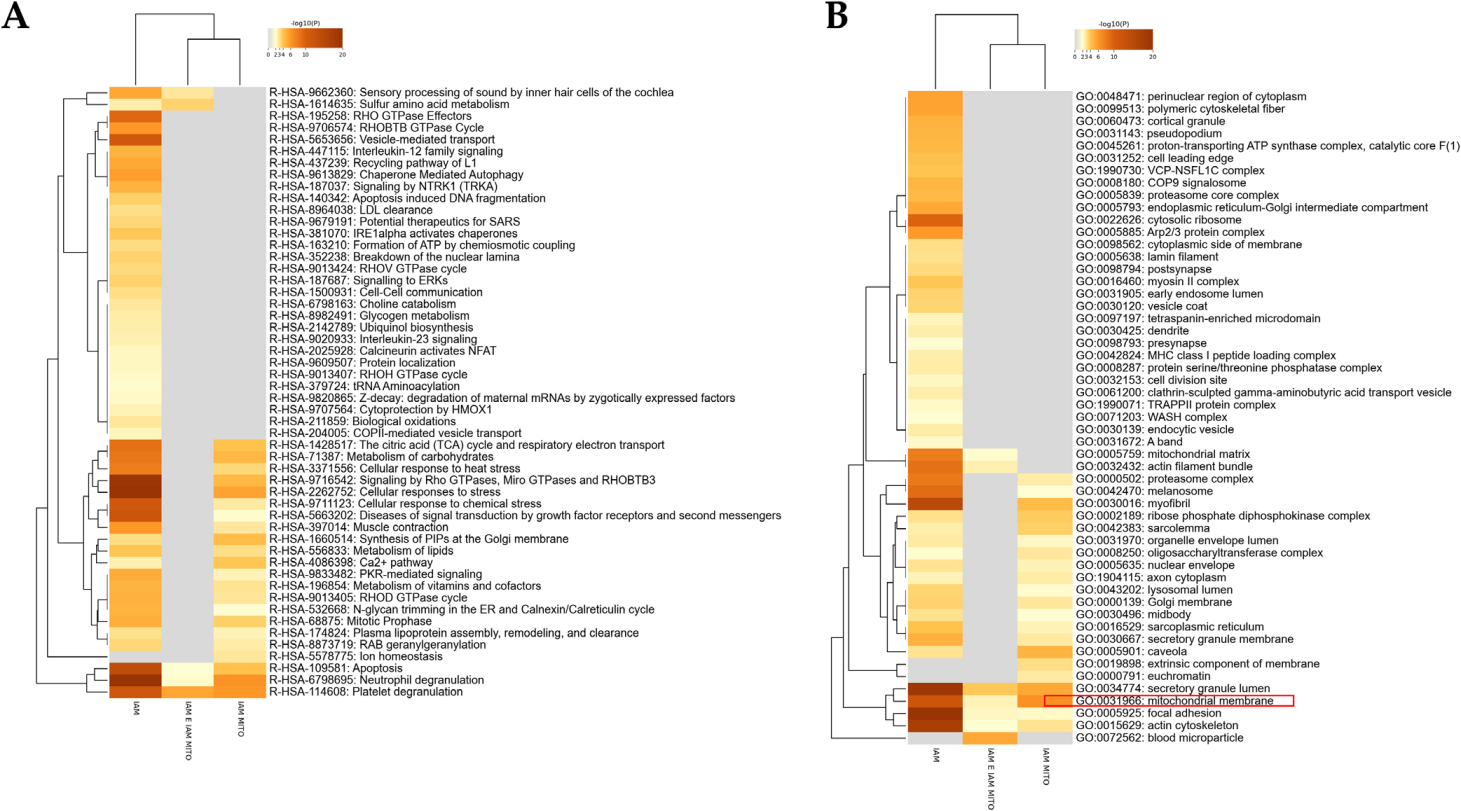
Functional impact of the deregulated proteostasis across MI and mitochondrial transplantation. (A) Functional mapping of the common pathways altered between the groups. (B) Functional mapping of disrupted proteome grading at subcellular level.

To analyze the differences observed in the proteome, datasets were functionally analyzed. Figure 6A shows the common pathways and processes altered between the groups evaluated. Interestingly, at a subcellular level, the altered proteins are presented in the mitochondrial membrane, mitochondrial matrix, focal adhesion, and actin cytoskeleton, among others (Figure 6B).

**FIGURE 7:**
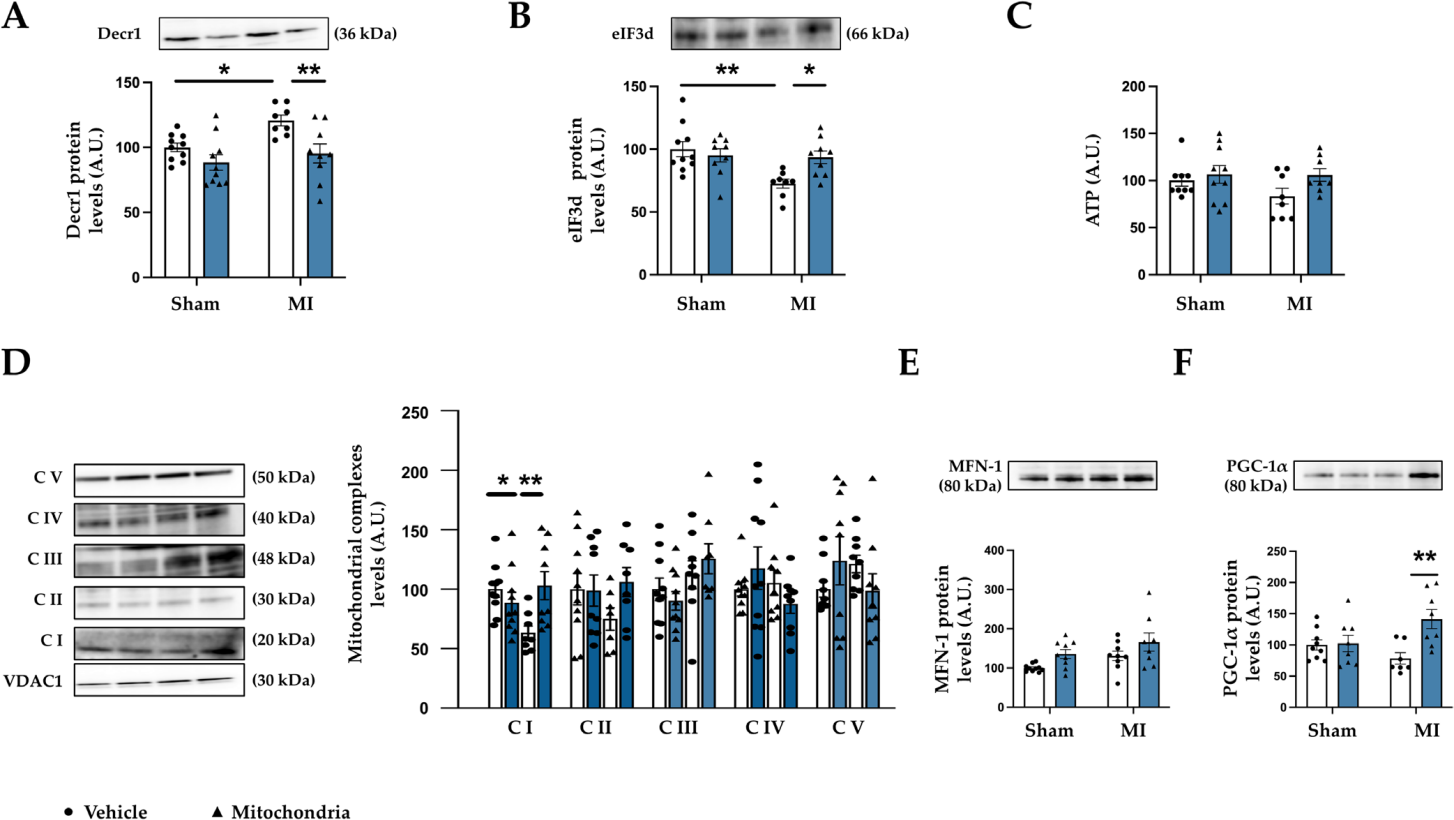
Mitochondrial transplantation improves mitochondrial alterations in MI animals. Protein levels of (A) 2,4‐dienoyl‐CoA Reductase 1 (Decr1); (B) eukaryotic translation initiation factor 3 subunit D (eIF3d); (C) Adenosine triphosphate levels (ATP); (D) mitochondrial complexes I to V (CI‐CV); (E) mitofusin‐1 (MFN‐1) and; (F) peroxisome proliferator‐activated receptor gamma coactivator 1‐alpha (PGC‐1α) in control rats (Sham) and rats submitted to myocardial infarction (MI). Bars graphs (blue: Mitochondrial transplantation; white: Respective controls) represent the means ± SEM of 7–10 animals with individual points for each rat. Statistics were performed using two‐way ANOVA followed by Bonferroni's multiple comparisons test. Protein levels were normalized by stain‐free or voltage‐dependent anion‐selective channel 1 (VDAC1). *p < 0.05; **p < 0.01; ***p < 0.001.

We validated and confirmed the data of proteomic analysis of 3 proteins that were modulated by both myocardial ischemia and mitochondrial transplantation. The selected proteins were Decr1 and eIF3d, two proteins involved in mitochondrial metabolism and Hmgb1, a mediator of inflammatory response. Myocardial ischemia was accompanied by an increase in levels of both Decr1 (Figure 7A) and Hmgb1 (Figure S4), although it was associated with a decrease in protein levels of eIF3d (Figure 7B). The administration of viable mitochondria prevented all these changes, confirming the results obtained by proteomic analysis.

MI‐animals showed non‐significant differences in ATP cardiac levels as compared to Sham animals, although the data does not reach statistical differences, which is recovered in animals that received viable mitochondria (Figure 7C). Mitochondrial transplantation was also able to prevent mitochondrial alterations observed in MI animals. In this sense, MI animals presented a decrease in mitochondrial complex I (Figure 7D) without modifications in any other complex or MFN‐1 protein levels (Figure 7E). Injection of viable mitochondria was able to prevent this alteration, an effect that was accompanied by an increase in the master regulator of mitochondrial biogenesis, PGC‐1α (Figure 7D–F).

### 3.6 Cardiac Mitochondrial Transplantation Prevents Renal Alterations in MI Animals

Finally, we explored if the beneficial effects of mitochondrial transplantation at the cardiac level could have any impact on other organs affected by MI. Based on the proteomic performed, an Ingenuity Pathway Analysis (IPA) was applied to the list of differentially expressed proteins obtained from the comparisons between MI and control animals and between MI and MI treated with mitochondrial transplantation animals. Pathway enrichment and network analyses were filtered to prioritize cardiovascular‐ and renal‐related biological functions. The most significantly represented canonical pathways were mainly related to cytoskeletal organization and cell–cell/cell–matrix interactions, including Rho GTPase‐dependent signaling, actin cytoskeleton signaling and integrin‐related pathways, as well as pathways associated with cardiac remodeling, such as dilated cardiomyopathy and cardiac hypertrophy signaling. In addition, pathways linked to inflammatory and immune responses and extracellular matrix and fibrosis‐related signaling were also significantly represented. Notably, several of these pathways showed an opposite or attenuated predicted activation pattern in MI animals treated with mitochondrial transplantation compared with untreated MI animals (Figure S5).

The heart and kidney are tightly interconnected through hemodynamic, neurohumoral, and inflammatory pathways, such that injury in one organ can directly trigger dysfunction in the other. Following MI, activation of the renin–angiotensin–aldosterone system and the systemic release of inflammatory mediators contribute to renal hypoperfusion, tubular injury, and progressive fibrosis—hallmarks of cardiorenal syndrome. Mitochondrial transplantation in infarcted animals was able to reduce plasma aldosterone levels compared with the untreated group (Table S4). Similarly, the levels of an inflammatory cytokine (CCL2) and an anti‐inflammatory cytokine (IL‐4) were measured. The data show that there were no changes in the plasma concentrations of either CCL2 or IL‐4 in any of the groups examined (Table S4). These results suggest that cardiac mitochondrial transplantation could promote an impact on renal tissue.

**FIGURE 8:**
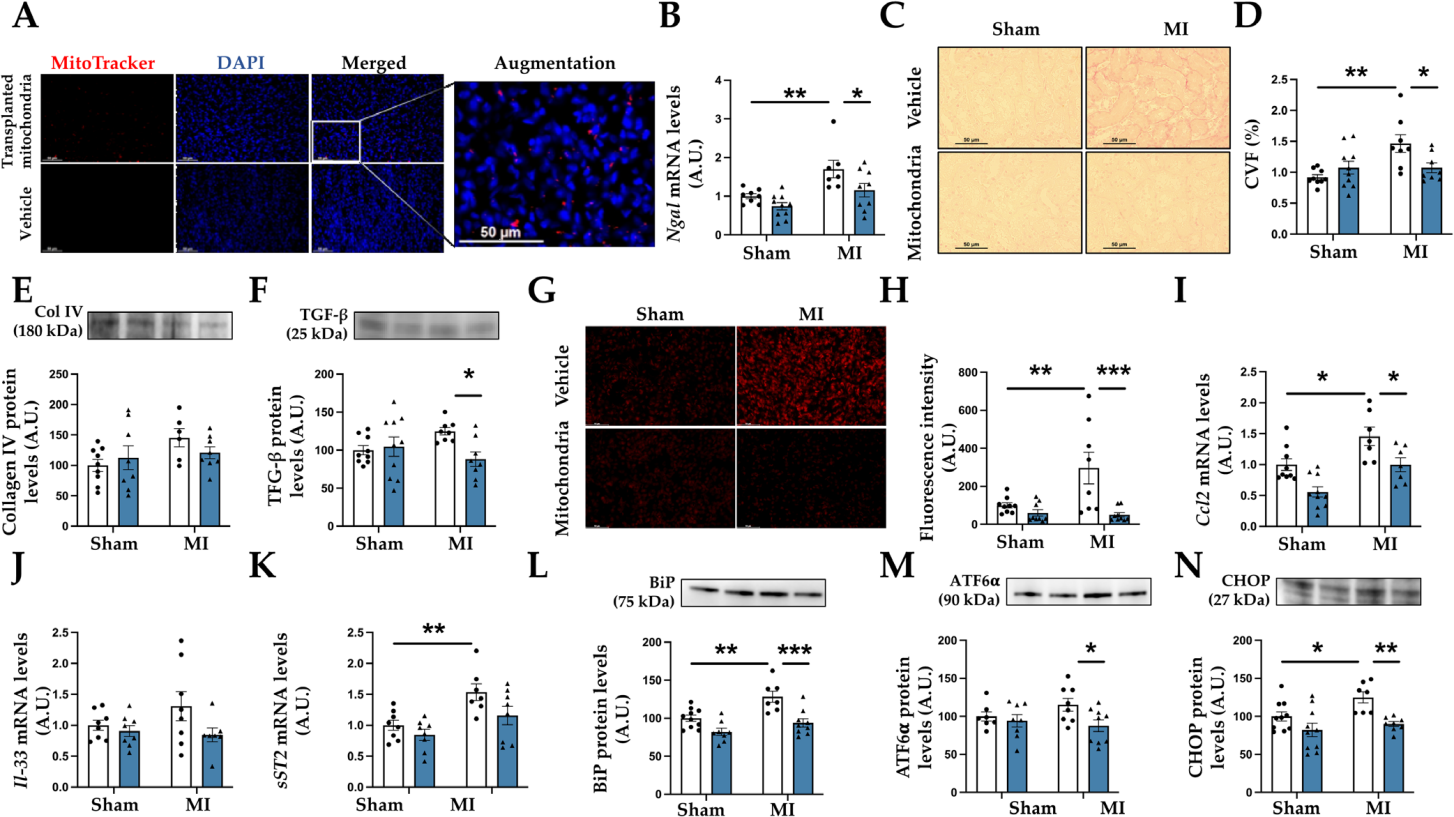
Mitochondrial transplantation improves renal alterations in MI animals. (A) Fluorescence images showing MitoTracker Deep Red FM–labeled transplanted mitochondria (red), nuclei stained with 4′,6‐diamidino‐2‐phenylindole (DAPI; blue), and merged views in renal tissue (F) from an animal that received labeled mitochondria (up) and from a Sham animal (down). (B) Gene expression of neutrophil gelatinase‐associated lipocalin (NGAL); (C) representative microphotographs of renal sections stained with picrosirius red (magnification 40×); and (D) quantification of collagen volume fraction (CVF). Protein levels of (E) collagen IV and (F) transforming growth factor‐β (TGF‐β). (G) Representative microphotographs of renal sections labeled with the oxidative dye dihydroethidium (magnification 40×). (H) Quantification of renal superoxide anion levels. Gene expression of (I) chemokine (C‐C motif) ligand 2 (CCL2); (J) interleukin‐33 (IL‐33) and (K) IL‐33 soluble receptor (sST2). Protein levels of (L) binding immunoglobulin protein (BiP); (M) activating transcription factor 6 alpha (ATF6α) and (N) CCAAT‐enhancer‐binding protein homologous protein (CHOP) in control rats (Sham) and rats submitted to myocardial infarction (MI). Bars graphs (blue: Mitochondrial transplantation; white: Respective controls) represent the means ± SEM of 7–10 animals with individual points for each rat. Statistics were performed using two‐way ANOVA followed by Bonferroni's multiple comparisons test. Data was normalized by stain‐free or 18S for protein levels or gene expression, respectively. *p < 0.05; **p < 0.01; ***p < 0.001.

Interestingly, renal samples revealed a positive fluorescent labeling in the kidney of the animals that received the transplantation of labeled mitochondria, whereas no signal was detected in Sham animals (Figure 8A) confirming the uptake of transplanted mitochondria. Infarcted animals showed an increase in NGAL, a marker of acute kidney injury (Figure 8B). We also observed an enhancement in renal interstitial fibrosis (Figure 8C,D), which was accompanied by a slight increase, but not significant, in collagen IV and TGF‐β protein levels (Figure 8E,F). Cardiac mitochondrial transplantation ameliorated these changes, preventing renal NGAL upregulation and fibrosis (Figure 8B–F). In addition, MI showed an oxidative stress environment suggested by the increase in superoxide anion levels observed in the kidneys of the animals (Figure 8G,H). These effects were accompanied by an increase in inflammatory markers, such as Ccl2 (Figure 8I) and sSt2 (Figure 8K) mRNA levels in the kidney of MI animals although no changes in IL33 (Figure 8J). Renal MI‐associated increases in inflammatory markers were avoided when animals were subjected to cardiac mitochondrial transplantation (Figure 8I–K). Finally, cardiac mitochondrial transplantation was able to prevent the activation of ER stress observed in kidney of MI animals characterized by an increase in the protein levels of BiP (Figure 8L), ATF6⍺ (Figure 8M), and CHOP (Figure 8N).

## 4 Discussion

The present study demonstrates that functional mitochondrial transplantation markedly attenuates chronic cardiac and renal alterations 4 weeks after myocardial infarction in male rats. Mitochondrial transplantation improved cardiac function and prevented the development of cardiac hypertrophy, while also limiting renal injury in infarcted animals. These protective effects are associated with reduced extracellular matrix synthesis, oxidative stress, inflammation, and endoplasmic reticulum stress activation, supporting mitochondrial transplantation as a potential therapeutic strategy to mitigate chronic MI‐related damage.

One of the critical steps in mitochondrial transplantation is to obtain pure and tightly coupled mitochondria. Isolated mitochondria contained mitochondrial proteins such as VDAC1, a protein located in the outer membrane of the mitochondria [23], and the mitochondrial complexes in charge of ATP production [24]. However, α‐tubulin, a cytoplasmic protein, was not detected in isolated mitochondria, denoting the absence of extramitochondrial proteins in the isolated extracts. In addition, the mitochondrial oxygen consumption rate was evaluated under pyruvate/malate and succinate substrates of complex I‐ and complex II‐linked substrates, respectively, in the presence of ADP. Under these conditions, high respiratory control ratios were registered, indicating that energy transduction in the mitochondrial preparations was well preserved.

Another important aspect to be considered is the estimation of mitochondrial dose. Several methods have been described to determine mitochondrial number, such as immunofluorescence staining, flow cytometry, or an indirect method, such as mitochondrial protein quantification. The direct methods allow us to estimate the number of mitochondria isolated. However, both methods present important disadvantages like the aggregation and overlapping of the mitochondria, which underestimates the number of mitochondria or the time needed for the quantification, a risk for mitochondrial integrity and viability [25]. Over recent years, the total amount of protein from isolated mitochondria rather than the number of mitochondria has been evaluated [26]. This indirect approach presents several advantages, such as the short time required, the wide availability of the technique, and its low cost.

In the last few years, mitochondrial transplantation has been evaluated due to the relevance of mitochondrial functions to the organism. Shin et al. demonstrated that mitochondrial transplantation did not alter hemodynamic parameters, heart rate, or cardiac rhythm at basal conditions, showing the safety of mitochondrial transplantation, also referred to as mitotherapy, in myocardial ischemia–reperfusion injury [7]. In this sense, previous studies have demonstrated that autologous mitochondrial transplantation does not elicit an autoimmune or adverse immunological response, as evidenced by the absence of antimitochondrial antibodies and reduced inflammatory activation following mitochondrial delivery [27]. In agreement with those observations, in the present study, no statistical differences were found after mitochondrial transplantation in non‐infarcted animals in any of the parameters evaluated. It has been demonstrated that direct injection of coupled mitochondria within the cardiac muscle promotes mitochondria uptake near the injection site area, promoting cardioprotection in the acute phase under ischemia–reperfusion conditions [28]. In accordance with that, we directly injected mitochondria at four different sites of the heart close to the ligature of the coronary artery at the moment of the MI induction. In addition, intracoronary delivery of mitochondria through the coronary arteries at the onset of the reperfusion phase shows a rapid distribution throughout the heart, decreasing infarct size and improving myocardial function [29]. Previous studies have demonstrated that exogenously delivered mitochondria can be internalized by cardiomyocytes through an active, actin‐dependent endocytic process. Following internalization, transplanted mitochondria have been shown to integrate functionally within host cells, contributing to improved cellular bioenergetics and metabolic support [30, 31]. The same results have been observed in other cell lines, such as neurons or skeletal myoblasts [32].

Different studies have demonstrated that autologous mitochondria transplantation promotes beneficial effects at the cardiac level, increasing LVEF, decreasing LV pressure and infarct size under acute conditions such as ischemia–reperfusion in different species [7, 8, 27, 28, 29, 33]. However, not so much information regarding the effects of mitochondrial transplantation in chronic conditions is available. In the current investigation, mitochondrial transplantation improved cardiac contractile and relaxing capacity in MI animals since they showed normal systolic and diastolic functions 4 weeks after transplantation and MI induction. This improvement in systolic function is shown not only by the amelioration in LVEF, but also in global longitudinal strain. This is a sensitive marker of myocardial dysfunction, since it reflects the function of sub‐endocardial longitudinally oriented fibers, which are more prone to ischemic damage and predict clinical outcomes and mortality in patients with HF [34, 35, 36]. Magnetic resonance studies revealed that transplanted animals, as compared to MI animals, did not develop cardiac hypertrophy, preserving normal cardiac structure. A reduction in myocardial fibrosis accompanied these functional effects. The beneficial effects of mitochondrial transplantation on cardiac fibrosis have been previously reported in different conditions. Thus, mitochondrial transplantation was able to prevent the development of cardiac fibrosis induced by chemotherapeutic drugs [37] and antibiotics [38]. It is well described that cardiac fibrosis promotes cardiomyocyte hypertrophy and cardiac stiffness, affecting myocardial function and promoting HF [39]. Mitochondrial transplantation prevented the increase in collagen I and in the profibrotic mediators CCN2 and TGF‐β 4 weeks post‐MI induction, showing its effects on ECM proteins, suggesting that this reduction could rely, at least in part, on a reduction in ECM synthesis.

Previous studies have demonstrated the positive effects of mitochondrial transplantation on oxidative stress in different situations, such as nephrotoxicity induced by doxorubicin [9], Parkinson's disease [40] or under ischemia–reperfusion in different tissues, including spinal cord [41] or brain [11]. It is well described that oxidative stress and inflammation are important triggers of fibrosis development. In agreement with that, we observed that mitochondrial transplantation prevented the MI‐related increase in superoxide anion levels. This effect seems to be independent of antioxidant defenses, since no changes were observed in SOD mRNA levels, suggesting that the superoxide anion variations might be related to changes in mitochondrial superoxide anion generation. Moreover, MI animals presented an important increase in inflammatory markers, which was reduced by mitochondrial transplantation. This result is in agreement with other studies in which mitochondrial transplantation improved the inflammatory status [27, 37, 42]. Regarding inflammation, the effects of mitotherapy on the IL‐33/ST2 axis are particularly notable. IL‐33 is a cytokine released under stress conditions to induce anti‐inflammatory and protective effects by binding to ST2L receptor [43]. However, under pathological conditions, there is an increase in the soluble receptor sST2, which acts as a decoy of IL‐33, promoting inflammation and oxidative stress. sST2 is produced by cardiac cells such as fibroblasts and myocytes, promoting aberrant collagen synthesis and inflammation [43]. Interestingly, it has been demonstrated that sST2 induces oxidative stress and mitochondrial damage and participates in cardiac pathologies such as aortic stenosis [44]. Due to these effects, sST2 has been proposed as a new biomarker of HF, since its levels increase in cardiovascular events independently of body mass, renal function, or age [45]. We show for the first time that mitochondrial transplantation avoids the increase in sST2 expression in MI animals, without affecting ST2L expression.

Previous studies in our group and others have demonstrated the role of ER stress in cardiac alterations [46, 47, 48, 49]. ER is an organelle responsible for protein synthesis and folding, essential for cell survival. An excessive accumulation of unfolded/misfolded proteins in the lumen of ER promotes the dissociation of BiP from different transmembrane proteins, initiating the unfolding protein response and activating the ER stress. The consequences of ER stress activation are the up‐regulation of different downstream proteins such as CHOP, ATF4, or ATF6α, among others, promoting apoptosis, inflammation, and tissue injury [50] if the situation is not resolved. It has been postulated that interventions focused on ER stress could serve as a potential therapeutic strategy for cardiac injury induced by ischemia and reperfusion [49]. However, the effects of mitochondrial transplantation on ER stress at the cardiac level have never been evaluated. The present study shows that mitochondrial transplantation ameliorated ER stress activation in MI animals. Consequently, this might contribute to the positive effects of mitotherapy on cardiac fibrosis and inflammation. These results agree with a previous study where mitochondrial transplantation improves ER stress in an animal model of spinal cord ischemia [41]. The connection between ER stress and mitochondria is well established since both organelles are connected through cholesterol‐rich microdomains, called MAM (mitochondria‐associated membranes), to coordinate cellular responses [51]. Altogether, these findings indicate that the beneficial effects of mitochondrial transplantation in chronic MI in male rats are primarily mediated through the modulation of oxidative stress, inflammatory signaling and ER stress responses, which are key drivers of adverse cardiac remodeling.

Proteomic analyses showed that the main altered proteins are present in the mitochondria, altering different pathways that could be involved in the cardiac consequences of MI. In the present study, we provide the proteome obtained from MI and mitochondrial transplanted animals, which could be useful in identifying novel mechanisms and key processes and providing a more detailed understanding of its potential as a treatment for post‐MI cardiac dysfunction. In order to further characterize the biological relevance of the proteomic alterations, an additional pathway and network analysis was performed using Ingenuity Pathway Analysis, considering separately the comparisons between MI and control animals and between MI and mitochondrial transplanted animals. The extended pathway and network analysis further supports the notion that cardiac mitochondrial transplantation modulates coordinated molecular programs involved in post‐infarction remodeling rather than isolated protein changes. The pathways most prominently affected were related to cytoskeletal organization and cell–matrix interactions, Rho GTPase‐dependent signaling, and integrin‐associated pathways, together with canonical pathways associated with cardiac remodeling, including dilated cardiomyopathy and cardiac hypertrophy signaling. In parallel, pathways linked to inflammatory responses and fibrosis‐related processes were also significantly represented. Importantly, the opposite or attenuated regulation of several of these functional modules in mitochondrial‐treated animals compared with untreated infarcted hearts is consistent with the reduced myocardial and renal structural damage observed in this study and supports a coordinated modulation of remodeling and inflammatory networks following mitochondrial transplantation. Among the proteins identified in proteomic analysis, Decr1 and eIF3d were validated. Decr1, a rate‐limiting enzyme in fatty‐acid β‐oxidation and highly expressed in cardiomyocytes, was upregulated after MI. This observation is consistent with previous reports showing that Decr1 overexpression exacerbates cardiac injury and dysfunction in ischemia [52, 53] and diabetic cardiomyopathy [54, 55] by disrupting mitochondrial metabolism. Mechanistically, Decr1 has been shown to reduce ATP availability and impair the activities of mitochondrial complexes I–IV, thereby promoting fibrosis and functional deterioration. In contrast, levels of eIF3d, a component of the translation initiation complex required for efficient synthesis of mitochondrial proteins [56]—particularly those involved in the electron transport chain—were reduced after MI. Decreased eIF3d expression has been associated with impaired mitochondrial respiration and increased oxidative stress [57, 58], further compromising mitochondrial homeostasis. Notably, mitochondrial transplantation normalized these alterations, and also with improved ATP content and increased PGC‐1α expression at the cardiac level, indicative of enhanced mitochondrial dynamics and function.

Previous studies have demonstrated the effects of mitochondrial transplantation at the renal level under acute pathologies [12, 26, 59]. We show for the first time that cardiac mitochondrial transplantation also prevented renal fibrosis, inflammation, oxidative stress, and ER stress in MI animals. These results have a potential significance, since it has been demonstrated that patients with renal alterations and cardiac damage have a worse prognosis than patients without renal impairment [60]. In previous studies, we have confirmed the detrimental role of MI at the renal level [61]. Cardiac mitochondrial transplantation attenuated renal injury secondary to myocardial infarction, indicating a beneficial effect beyond the myocardium. This renoprotective effect is likely related to the ability of mitochondrial transplantation to attenuate early pathological processes triggered by MI, particularly oxidative stress and inflammation, which are major contributors to tissue damage and multi‐organ dysfunction. Preservation of cardiac structure and function may further limit systemic stress signals that promote secondary renal injury. Nevertheless, the precise molecular mechanisms underlying heart–kidney communication in this context were not directly addressed in the present study and remain to be fully elucidated. Elucidating these pathways represents an important direction for future research. In this context, circulating aldosterone levels were elevated in infarcted animals, although they did not reach statistical significance. Mitochondrial transplantation in infarcted animals was able to reduce plasma aldosterone levels compared with the untreated group.

In addition to the promising results obtained in animal models, the translational potential of mitochondrial transplantation has been supported by a preliminary clinical study in pediatric patients with cardiogenic shock following ischemia–reperfusion injury, in which autologous mitochondrial administration improved ventricular function allowing the separation from extracorporeal membrane oxygenation and a ventricular function improvement, as compared with those patients undergoing revascularization alone [62]. However, despite these encouraging findings, this pilot study could not address relevant issues, including efficacy, immune responses, or the standardization of procedures. Such issues are necessary to be addressed before this approach can be advanced toward broader clinical application of mitochondrial transplantation. First, methodological heterogeneity remains a major limitation: studies differ in the source of mitochondria, isolation protocols, and the techniques used to quantify mitochondrial yield, purity, and functional integrity. Establishing standardized procedures for mitochondrial isolation and quantification will be essential for ensuring the treatment in the clinical setting. Second, the optimal route of delivery has not been defined. While direct intramyocardial injection is effective in experimental models, alternative strategies—such as intracoronary infusion, minimally invasive catheter‐mediated delivery, and systemic administration—may offer greater clinical feasibility and should be systematically evaluated for safety, biodistribution, and therapeutic efficacy. Finally, immune response is another critical point that needs to be deeply explored. While transplantation of autologous mitochondria seems to be safe without inflammation and autoimmune responses for cardioprotection in acute conditions in different models [27, 28, 63], as well as in the already mentioned clinical setting [62], the immune responses generated by allogeneic mitochondrial transplantation are not well established, since it has been shown that single or serial injections of either syngeneic or allogeneic mitochondria were unable to significantly trigger either short‐ or long‐term direct or indirect immune responses in mice [64]. However, the acute administration of allogenic mitochondria does lead to immune response and heart rejection in mice [65]. Therefore, resolution of immunological challenges, as well as other issues, is required for expanding the potential of mitochondrial transplantation as a therapeutic option.

## 5 Conclusion

This study demonstrates that cardiac mitochondrial transplantation is an effective therapeutic strategy in a chronic model of MI, extending the evidence for this intervention beyond the acute ischemia–reperfusion settings previously reported. By administering functional mitochondria directly into the myocardium at the time of MI induction and evaluating outcomes 4 weeks later, we provide novel proof of long‐term cardioprotective efficacy. Mitochondrial transplantation preserved LV function, reduced cardiac hypertrophy, and attenuated interstitial fibrosis, effects that were accompanied by decreases in oxidative stress, inflammation, and ER stress activation.

Importantly, our findings also reveal significant renal protection in male subjects, an aspect that has been minimally explored in earlier cardiac mitochondrial transplantation studies. The attenuation of renal injury in this chronic MI context suggests that mitochondrial therapy may exert additional benefits, potentially impacting key mechanisms of cardiorenal interaction.

Overall, this work highlights mitochondrial transplantation in males as a promising therapeutic approach capable of conferring sustained protection to both the heart and kidney after permanent MI. However, it is necessary to validate whether these beneficial effects are independent of gender. These results support further investigation of mitochondrial therapy as a candidate intervention for chronic cardiovascular disease and cardiorenal syndromes.

## 6 Limitations

This study has several limitations that should be acknowledged. First, we did not assess whether the therapeutic efficacy of mitochondrial transplantation varies across different severities of MI. Because infarct size can influence both cardiac remodeling and downstream renal consequences, future studies should evaluate whether the magnitude of benefit is preserved in mild, moderate, and large infarcts.

Second, although our findings indicate sustained functional improvement 4 weeks after treatment, we did not investigate long‐term safety aspects of mitochondrial transplantation. Potential concerns such as immune responses to exogenous mitochondria, unintended mitochondrial persistence, or excessive cellular proliferation were not evaluated and will require dedicated long‐term follow‐up studies.

Third, in the present study, we provide the cardiac proteome obtained from MI and mitochondrial transplanted animals, which could be useful in identifying novel mechanisms and key processes and providing a more detailed understanding of its potential as a treatment for post‐MI cardiac dysfunction. However, proteomic or transcriptomic analyses of renal tissue in future work would provide a more comprehensive understanding of cross‐organ regulatory mechanisms associated with mitochondrial therapy.

Finally, in this study all the experiments were performed exclusively in male rats. Sex differences can significantly influence mitochondrial function and cardiac and renal responses to ischemic injury. In particular, estrogens have been shown to regulate mitochondrial biogenesis, respiratory capacity, and susceptibility to injury, which may affect the efficacy of mitochondrial transplantation. Future studies including female animals are warranted to evaluate potential sex‐specific effects and to increase the translational applicability of mitochondrial transplantation therapies.

## Author Contributions

M.C.‐C., A.M.‐G., A.R.‐M., B.D.‐V., S.J.‐G., R.R.‐D., V.C. and E.M.‐M. performed experiments and analyzed the data. F.I. performed cardiac functional evaluation. B.R. performed MI induction. M.C.‐C., A.M.‐G. and R.G. performed mitochondrial isolation and functional evaluation. J.F.‐I. and E.S. performed proteomic studies and analysis. F.I., B.R., M.L.‐N., V.C. and E.M.‐M. conceived, supervised the project and wrote the manuscript, and all authors revised the manuscript and approved its final version.

## Funding

This work was supported by Instituto de Salud Carlos III‐Fondo Europeo de Desarrollo Regional (FEDER) (PI21/00431 and CIBERCV). M.C.‐C. was supported by a contract from Universidad Complutense de Madrid y Banco Santander (CT15/23). A.M.‐G. was supported by a contract from CAM (Ayuda de empleo juvenil CT41/22/PEJ‐2021‐AI/BMD‐22002).

## Conflicts of Interest

The authors declare no conflicts of interest.

## Supporting information


**Figure S1:** Effects of mitochondrial transplantation on extracellular matrix and oxidative stress markers in MI animals. Effects of mitochondrial transplantation on gene expression of (A) collagen I (*Col1a1*); (B) cellular communication network factor 2 (*Ccn2*); (C) transforming growth factor‐β (*Tgf‐β*); (D) fibronectin; (E) superoxide 1 (*Sod1*); (F) superoxide 2 (*Sod2*) and (G) IL‐33 ligand receptor (*St2l*) in control rats (Sham) and rats submitted to myocardial infarction (MI). Bars graphs (blue: mitochondrial transplantation; white: respective controls) represent the means ± SEM of 7–10 animals with individual points for each rat. Statistics were performed using two‐way ANOVA followed by Bonferroni's multiple comparisons test. Gene expression was normalized by 18S. *p < 0.05; ***p < 0.001.
**Figure S2:** Hierarchical clustering heatmap of differentially expressed cardiac proteins. The heatmap displays normalized protein abundance values, with bootstrap‐supported clustering shown on the sample dendrogram.
**Figure S3:** Functional impact of the deregulated proteostasis across MI animals. (A) Functional mapping of the common pathways altered and (B) functional mapping of disrupted proteome grading at subcellular level between myocardial infarction (MI) and control animals (Sham).
**Figure S4:** Protein levels of High mobility group box 1 (Hmgb1) in control rats (Sham) and rats submitted to myocardial infarction (MI). Bars graphs (blue: mitochondrial transplantation; white: respective controls) represent the means ± SEM of 8–10 animals with individual points for each rat. Statistics were performed using two‐way ANOVA followed by Bonferroni's multiple comparisons test. Protein levels were normalized by stain‐free, and gene expression was normalized by 18S. *p < 0.05; **p < 0.01.
**Figure S5:** Predictive activation profile of pathways. Based on proteomics datasets, Ingenuity Pathway Analysis (IPA) software was used to obtain the activation prediction of significantly altered pathways. The comparisons were performed between MI and control animals and between MI and MI treated with mitochondrial transplantation animals. Blue and orange colors indicate inhibition and activation directionality, respectively. Pathway enrichment analysis was filtered to prioritize cardiovascular‐ and renal‐related biological functions.
**Figure S6:** Original blots from (A) Figure 1; (B) Figure 3; (C) Figure 4 and (D) Figure 7 and (E) Figure 8.
**Table S1:** List of primers used in the study.
**Table S2:** Effects of mitochondrial transplantation on body weight, cardiac structure, systolic blood pressure (SBP) and infarct size in control rats (Sham) and rats submitted to myocardial infarction (MI).

**Table S3:** Proteomic dataset of cardiac tissue.
**Table S4:** Effects of mitochondrial transplantation on plasma levels of aldosterone and inflammatory markers in control rats (Sham) and rats submitted to myocardial infarction (MI).

## Data Availability

The authors are willing to provide the raw data generated in the study upon reasonable request.
