# Supplementary material for: Mitochondrial Transplantation as a New Therapeutic Approach Against Cardiac and Renal Consequences in Male Rats With Myocardial Infarction
Source: Acta Physiol (Oxf). 2026 Apr 25;242:e70231. doi: 10.1111/apha.70231 (PMC13109771; doi:10.1111/apha.70231)
Supplement: Supplementary file 1 — Figure S1: Effects of mitochondrial transplantation on extracellular matrix and oxidative stress markers in MI animals. Effects of mitochondrial transplantation on gene expression of (A) collagen I (Col1a1); (B) cellular communication network factor 2 (Ccn2); (C) transforming growth factor‐β (Tgf‐β); (D) fibronectin; (E) superoxide 1 (Sod1); (F) superoxide 2 (Sod2) and (G) IL‐33 ligand receptor (St2l) in control rats (Sham) and rats submitted to myocardial infarction (MI). Bars graphs (blue: mitochondrial transplantation; white: respective controls) represent the means ± SEM of 7–10 animals with individual points for each rat. Statistics were performed using two‐way ANOVA followed by Bonferroni's multiple comparisons test. Gene expression was normalized by 18S. *p < 0.05; ***p < 0.001. Figure S2: Hierarchical clustering heatmap of differentially expressed cardiac proteins. The heatmap displays normalized protein abundance values, with bootstrap‐supported clustering shown on the sample dendrogram. Figure S3: Functional impact of the deregulated proteostasis across MI animals. (A) Functional mapping of the common pathways altered and (B) functional mapping of disrupted proteome grading at subcellular level between myocardial infarction (MI) and control animals (Sham). Figure S4: Protein levels of High mobility group box 1 (Hmgb1) in control rats (Sham) and rats submitted to myocardial infarction (MI). Bars graphs (blue: mitochondrial transplantation; white: respective controls) represent the means ± SEM of 8–10 animals with individual points for each rat. Statistics were performed using two‐way ANOVA followed by Bonferroni's multiple comparisons test. Protein levels were normalized by stain‐free, and gene expression was normalized by 18S. *p < 0.05; **p < 0.01. Figure S5: Predictive activation profile of pathways. Based on proteomics datasets, Ingenuity Pathway Analysis (IPA) software was used to obtain the activation prediction of significantly altered pathways. The [file APHA-242-e70231-s001.docx]

**Supplemental material**

**Mitochondrial transplantation as a new therapeutic approach against cardiac and renal consequences in male rats with myocardial infarction**

María Cuesta-Corral^1†^, Alejandro Montoro-Garrido^1†^, Ana Romero-Miranda^1^, Fabián Islas^2^, Bunty Ramchandani^3^, Ricardo Gredilla^1^, Joaquín Fernández-Irigoyen^4^, Enrique Santamaría^4^, Beatriz Delgado-Valero^1^, Sara Jiménez-González^1^, Raquel Rodrigues Díez^1,5^, María Luisa Nieto^5,6^, Victoria Cachofeiro^1,5^*, Ernesto Martínez-Martínez^1,5*^.

^1^Departamento de Fisiología, Facultad de Medicina, Instituto de Investigación Sanitaria Gregorio Marañón (IiSGM), Universidad Complutense de Madrid, Madrid, Spain. ^2^Unidad de Imagen Cardíaca, Hospital General Universitario de Talavera de la Reina, Toledo, Spain. ^3^Servicio de Cirugía Cardiaca Infantil, Hospital La Paz, Madrid, Spain. ^4^Proteomics Platform, Navarrabiomed, Hospital Universitario de Navarra (HUN), Universidad Pública de Navarra (UPNA), IdiSNA, Pamplona, Spain. ^5^Ciber de Enfermedades Cardiovasculares (CIBERCV), Instituto de Salud Carlos III, Madrid, Spain. ^6^Instituto de Biología y Genética Molecular, CSIC-Universidad de Valladolid, Spain.

^†^Both authors contributed equally to this work.

^*^Both authors contributed equally to this study.

**Corresponding authors:**

Dr. Victoria Cachofeiro

Departamento de Fisiología, Facultad de Medicina de la Universidad Complutense de Madrid. Avenida Complutense s/n, 28040 Madrid, Spain. Tel: +34913941489. Fax: +34913941628. E-mail: [vcara@ucm.es](mailto:vcara@ucm.es)

Dr. Ernesto Martínez-Martínez

Departamento de Fisiología, Facultad de Medicina de la Universidad Complutense de Madrid. Avenida Complutense s/n, 28040 Madrid, Spain. Tel: +34913941489. Fax: +34913941628. E-mail: [ernmarti@ucm.es](mailto:ernmarti@ucm.es)

This file includes:

3 supplemental tables

6 supplemental figures

**Table S1.** List of primers used in the study.

| **Gene** | **Primer** | **Sequence (5´to 3´)** |
| --- | --- | --- |
| ***18s*** | Forward | CATTCGAACGTCTGCCCTAT |
|  | Reverse | GTTTCTCAGGCTCCCTCTCC |
| ***Ccl2*** | Forward | TTCCTTATTGGGGTCAGCAC |
|  | Reverse | CAGTTAATGCCCCACTCACC |
| ***Col1a1*** | Forward | GCCTCCCAGAACATCACCTA |
|  | Reverse | ATGTCTGTCTTGCCCCAAGT |
| ***Ccn2*** | Forward | GAGTCGTCTCTGCATGGTCA |
|  | Reverse | CCACAGAACTTAGCCCGGTA |
| ***Fibronectin*** | Forward | GGGGTCACGTACCTCTTCAA |
|  | Reverse | TGGAGGTTAGTGGGAGCATC |
| ***Il-33*** | Forward | TGGCCTCACCATAAGAAAGG |
|  | Reverse | GCAAACGCTTGGATACTGC |
| ***Ngal*** | Forward | CGATGAACTGAAGGAGCGAT |
|  | Reverse | TCTGGCAACAGGAAAGATGG |
| ***Sod1*** | Forward | TAACTGAAGGCGAGCATGGG |
|  | Reverse | CCTCTCTTCATCCGCTGGAC |
| ***Sod2*** | Forward | CGGGGGCCATATCAATCACA |
|  | Reverse | TAGCCTCCAGCAACTCTCCT |
| ***sSt2*** | Forward | CGTTACCTTCCTGTGCCATT |
|  | Reverse | CTCCATTTGCCAATCATGTG |
| ***ST2L*** | Forward | AGTTGTGCATTTACGGGAGAG |
|  | Reverse | GGATACTGCTTTCCACCACAG |
| ***Tgf-β*** | Forward | CAGAAGTTGGCATGGTAGCC |
|  | Reverse | TGCTTCAGCTCCACAGAGAA |
| ***Tlr4*** | Forward | GCTTGAATCCCTGCATAGAGG |
|  | Reverse | TGTCTCCACAGCCACCAGATTCTC |

*Ccl2:* chemokine (C-C motif) ligand 2; *Col1a1*: collagen type I; *Ccn2:* cellular communication network factor 2; *Il-33*: interleukin-33; *Ngal:* neutrophil gelatinase-associated lipocalin; *Sod*: superoxide dismutase; *Tgf-β*: Transforming growth factor-beta and *Tlr4:* toll-like receptor 4.

**Table S2.** Effects of mitochondrial transplantation on body weight, cardiac structure, systolic blood pressure (SBP) and infarct size in control rats (Sham) and rats submitted to myocardial infarction (MI).

|  | **Sham** | **Sham**  **mitochondria** | **MI** | **MI**  **mitochondria** |
| --- | --- | --- | --- | --- |
| **Body weight (g)** | 342.44 ± 4.73 | 345.02 ± 8.33 | 332.64 ± 8.21 | 346.76 ± 8.33 |
| **SBP (mmHg)** | 123.60 ± 1.82 | 126.05 ± 1.69 | 126.10 ± 1.95 | 124.39 ± 1.52 |
| **LV mass (g)** | 0.336 ± 0.02 | 0.390 ± 0.016 | **0.412 ± 0.022*** | 0.345 ± 0.028 |
| **IVTSd (cm)** | 0.165 ± 0.007 | 0.156 ± 0.003 | 0.169 ± 0.004 | **0.150 ± 0.004^†^** |
| **IVTSs (cm)** | 0.286 ± 0.007 | 0.272 ± 0.010 | 0.278 ± 0.010 | 0.262 ± 0.007 |
| **EDD (cm)** | 0.628 ± 0.018 | 0.650 ± 0.014 | **0.686 ± 0.016**** | **0.632 ± 0.016^†^** |
| **ESD (cm)** | 0.356 ± 0.017 | 0.389 ± 0.014 | 0.380 ± 0.020 | 0.366 ± 0.016 |
| **PWT (cm)** | 0.158 ± 0.007 | 0.163 ± 0.005 | 0.161 ± 0.004 | 0.158 ± 0.005 |
| **FS (%)** | 43.16 ± 2.05 | 40.15 ± 1.31 | 42.74 ± 1.58 | 42.09 ± 1.65 |
| **Infarct size / LV mass (%)** | - | - | 18.68 ± 1.58 | 19.95 ± 1.77 |

SBP: systolic blood pressure; LV: left ventricle; IVTSd: interventricular septum end-diastolic thickness; IVTSs: interventricular septum end-systolic thickness; EDD: end-diastolic diameter; ESD: end-systolic diameter; PWT: posterior wall thickness; FS: fractional shortening. *p<0.05; **p<0.01 vs. Sham group; †p<0.05 vs. MI. Statistics were performed using two-way ANOVA followed by Bonferroni´s multiple comparisons test.

**Table S4.** Effects of mitochondrial transplantation on plasma levels of aldosterone and inflammatory markers in control rats (Sham) and rats submitted to myocardial infarction (MI).

|  | **Sham** | **Sham**  **mitochondria** | **MI** | **MI**  **mitochondria** |
| --- | --- | --- | --- | --- |
| **ALDO (A.U.)** | 100 ± 16.37 | 144.82 ± 18.88 | 139.52 ± 21.46 | **81.10 ± 9.40^†^** |
| **CCL2 (A.U.)** | 100 ± 8.05 | 140.24 ± 7.69 | 113.78 ± 10.55 | 116.42 ± 10.43 |
| **IL-4 (A.U.)** | 100 ± 8.05 | 77.95 ± 24.19 | 106.13 ± 33.11 | 86.16 ± 39.36 |

Aldo: aldosterone; CCL2: chemokine (C-C motif) ligand 2 and; IL-4: interleukin 4. †p<0.05 vs. MI. Statistics were performed using two-way ANOVA followed by Bonferroni´s multiple comparisons test.

**Figure S1. Effects of mitochondrial transplantation on extracellular matrix and oxidative stress markers in MI animals.** Effects of mitochondrial transplantation on gene expression of (**A**) collagen I (*Col1a1*); (**B**) cellular communication network factor 2 (*Ccn2*); (**C**) transforming growth factor-β (*Tgf-β*); (**D**) fibronectin; (**E**) superoxide 1 (*Sod1*); (**F**) superoxide 2 (*Sod2*) and (**G**) IL-33 ligand receptor (*St2l*) in control rats (Sham) and rats submitted to myocardial infarction (MI). Bars graphs (blue: mitochondrial transplantation; white: respective controls) represent the means ± SEM of 7–10 animals with individual points for each rat. Statistics were performed using two-way ANOVA followed by Bonferroni´s multiple comparisons test. Gene expression was normalized by 18S. *p< 0.05; ***p<0.001.

**Figure S2:** Hierarchical clustering heatmap of differentially expressed cardiac proteins. The heatmap displays normalized protein abundance values, with bootstrap-supported clustering shown on the sample dendrogram.

**Figure S3. Functional impact of the deregulated proteostasis across MI animals.** (**A**) Functional mapping of the common pathways altered and (**B**) functional mapping of disrupted proteome grading at subcellular level between myocardial infarction (MI) and control animals (Sham).

**Figure S4:** Protein levels of High mobility group box 1 (Hmgb1) in control rats (Sham) and rats submitted to myocardial infarction (MI). Bars graphs (blue: mitochondrial transplantation; white: respective controls) represent the means ± SEM of 8–10 animals with individual points for each rat. Statistics were performed using two-way ANOVA followed by Bonferroni´s multiple comparisons test. Protein levels were normalized by stain-free, and gene expression was normalized by 18S. *p<0.05; **p<0.01.


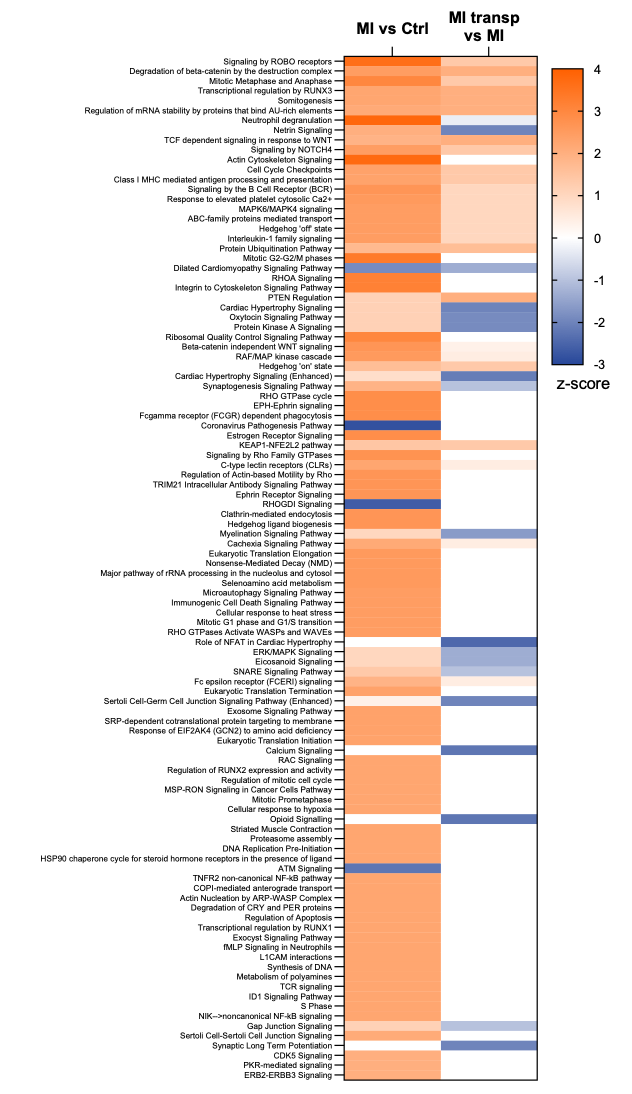


**Figure S5: Predictive activation profile of pathways.** Based on proteomics datasets, Ingenuity Pathway Analysis (IPA) software was used to obtain the activation prediction of significantly altered pathways. The comparisons were performed between MI and control animals and between MI and MI treated with mitochondrial transplantation animals. Blue and orange colors indicate inhibition and activation directionality, respectively. Pathway enrichment analysis was filtered to prioritize cardiovascular- and renal-related biological functions.

**A**

**B**

**C**

**D**

**E**

**Figure S6.** Original blots from (**A**) Figure 1; (**B**) Figure 3; (**C**) Figure 4 and (**D**) Figure 7 and (**E**) Figure 8.
